# Advancing the Therapeutic Potential of Indoleamides for Tuberculosis

**DOI:** 10.1128/AAC.00343-19

**Published:** 2019-06-24

**Authors:** Shichun Lun, Rokeya Tasneen, Tridib Chaira, Jozef Stec, Oluseye K. Onajole, Tian J. Yang, Christopher B. Cooper, Khisi Mdluli, Paul J. Converse, Eric L. Nuermberger, V. Samuel Raj, Alan Kozikowski, William R. Bishai

**Affiliations:** a Center for Tuberculosis Research, Department of Medicine, Johns Hopkins University School of Medicine, Baltimore, Maryland, USA; b Center for Drug Design Discovery and Development (C4D), SRM University, Delhi NCR, Sonepat, Haryana, India; c Department of Pharmaceutical Sciences, College of Pharmacy, Marshall B. Ketchum University, Fullerton, California, USA; d Department of Biological, Chemical and Physical Sciences, Roosevelt University, Chicago, Illinois, USA; e Global Alliance for TB Drug Development (TB Alliance), New York, New York, USA; f StarWise Therapeutics LLC, University Research Park, Madison, Wisconsin, USA

**Keywords:** MmpL3 inhibitor, *Mycobacterium tuberculosis*, chemotherapy, indole-2-carboxamide, mouse model, mycolic acid

## Abstract

Indole-2-carboxamide derivatives are inhibitors of MmpL3, the cell wall-associated mycolic acid transporter of Mycobacterium tuberculosis. In the present study, we characterized indoleamide effects on bacterial cell morphology and reevaluated pharmacokinetics and *in vivo* efficacy using an optimized oral formulation. Morphologically, indoleamide-treated M. tuberculosis cells demonstrated significantly higher numbers of dimples near the poles or septum, which may serve as the mechanism of cell death for this bactericidal scaffold. Using the optimized formulation, an expanded-spectrum indoleamide, compound 2, showed significantly improved pharmacokinetic (PK) parameters and *in vivo* efficacy in mouse infection models. In a comparative study, compound 2 showed superior efficacy over compound 3 (NITD-304) in a high-dose aerosol mouse infection model. Since indoleamides are equally active on drug-resistant M. tuberculosis, these findings demonstrate the therapeutic potential of this novel scaffold for the treatment of both drug-susceptible and drug-resistant tuberculosis.

## Abstract (precis)

Indole-2-carboxamide derivatives are inhibitors of MmpL3, the cell wall-associated mycolic acid transporter of Mycobacterium tuberculosis. In the present study, we characterized indoleamide effects on bacterial cell morphology and reevaluated pharmacokinetics and *in vivo* efficacy using an optimized oral formulation.

## INTRODUCTION

Despite decades of efforts and progress in tuberculosis (TB) control programs and research for new drugs and therapeutic regimens, TB continues to pose a great risk to global public health security as the leading cause of death by a single infectious agent. In 2016, an estimated 10.4 million new cases of TB occurred globally, including 600,000 cases with resistance to rifampin, the most powerful first-line TB drug in use (1). Also in 2016, there were 1.7 million deaths from TB globally, including 0.4 million deaths among people coinfected with HIV (1). Drug resistance has become more prominent because of the high global burden and poor treatment success rate (1, 2).

MmpL3 is an essential inner membrane-anchored protein of Mycobacterium tuberculosis, which transports trehalose monomycolate from the cytoplasm to the cell wall in the mycolic acid biosynthesis pathway (3–5). MmpL3 also plays a role in heme transport and iron acquisition (6, 7). As iron acquisition is essential for M. tuberculosis, the role of MmpL3 in heme transport further justifies MmpL3 as a viable drug target (8, 9). Indeed, multiple structural scaffolds of MmpL3 inhibitors with anti-TB activity have been discovered, including indoleamides (10, 11), the adamantyl urea AU1235 (4), SQ109 (12), the pyrrole derivative BM212 (13), and several other structural chemotypes (8, 14–16).

**FIG 1:**
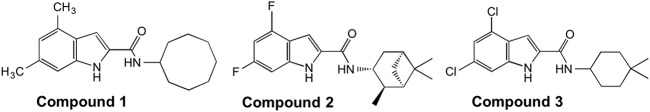
Chemical structures of compound 1 (designated compound 3 in reference 18), compound 2 (designated compound 26 in reference 19), and compound 3 (designated compound NITD-304 in reference 17).

Indoleamides have demonstrated extremely low MICs against both drug-susceptible and drug-resistant M. tuberculosis and activity in mouse infection models (17, 18). Indoleamides are bactericidal against M. tuberculosis (17, 18), most likely because of damage to the cell wall due to the lack of matured mycolic acids. However, the effect of indoleamide treatment on cell morphology has not been reported. Most interestingly, an expanded-spectrum indoleamide (Fig. 1, compound 2) showed significant synergy with rifampin both *in vitro* and in a mouse infection model (19), making this chemotype even more promising as a new TB drug candidate. Our objectives for this study were to determine the effect of indoleamide treatment on the cell morphology of M. tuberculosis and improve the pharmacokinetic (PK) profile and *in vivo* efficacy of the lead compound by optimizing the formulation.

## RESULTS

### Indoleamide treatment-induced morphological changes of M. tuberculosis.

**FIG 2:**
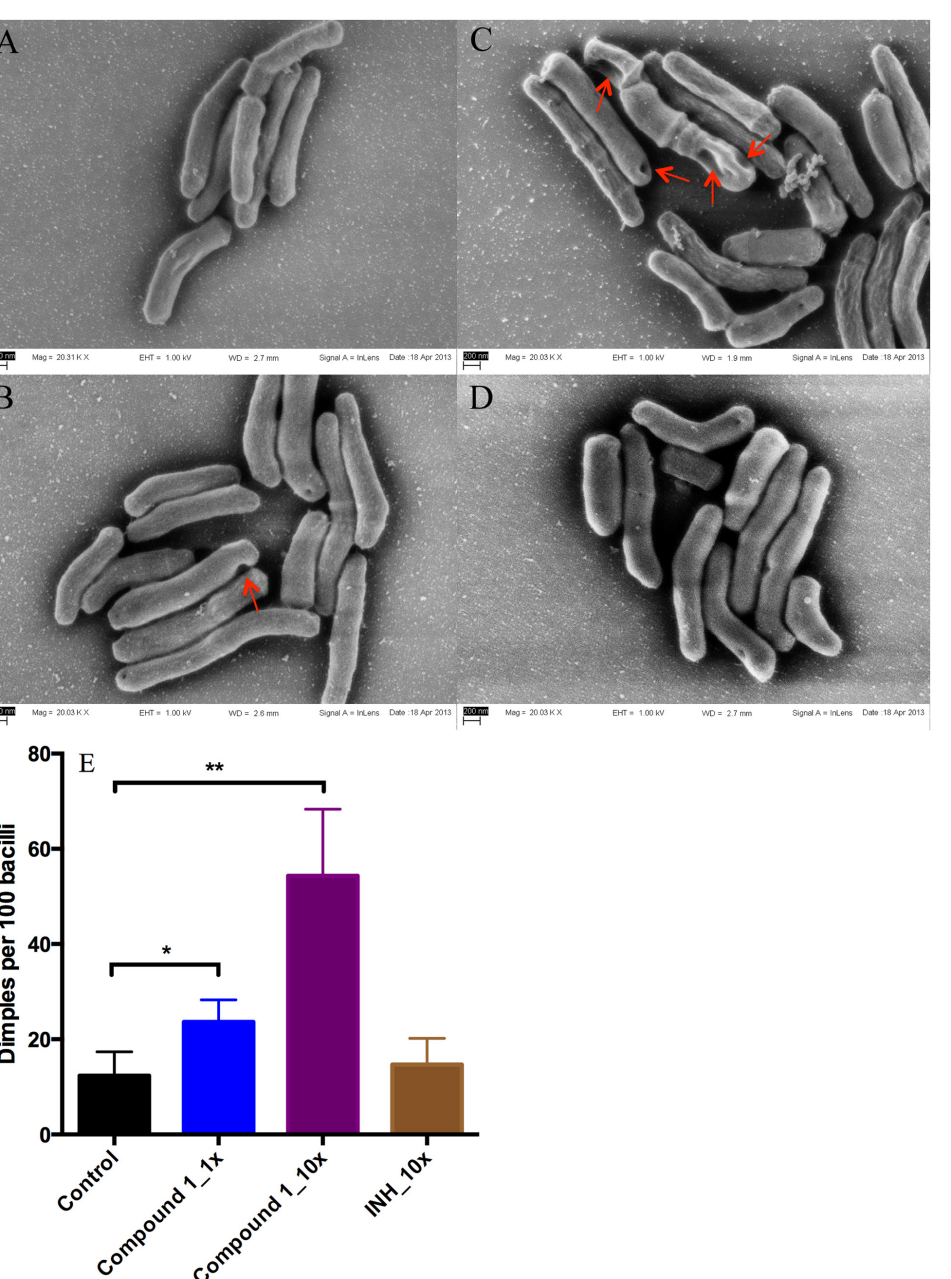
Morphological characterization of M. tuberculosis H37Rv after treatment with compound 1 or isoniazid (INH). (A) Untreated control; (B), treated with 1× MIC of compound 1; (C) treated with 10× MIC of compound 1; (D) treated with 10× MIC of INH (control); (E) number of dimples per 100 bacilli counted from three biological replicates. (mean ± SD; *P < 0.05, **P < 0.01 by Student’s *t* test).

To determine whether indoleamide treatment affects cell morphology, we treated mid-log-phase M. tuberculosis H37Rv cells with 1× and 10× MIC of compound 1 (18) and used untreated cells and cells treated with 10× MIC of isoniazid as controls. After 24 hours of treatment, cells were processed and subjected to field emission scanning electron microscopy (FESEM). Cells treated with compound 1 showed a significantly higher number of unhealthy cells, as exemplified by more dimples at the poles and septum and a wrinkled cell surface near the poles (Fig. 2A to D). Treatment with compound **1** at 10× MIC resulted in a higher rate of deformed bacilli than 1× MIC, suggesting a dose-dependent effect (Fig. 2E). Treatment with isoniazid (INH) at 10× MIC resulted in more dimples per 100 cells than no treatment, but the difference did not reach statistical significance (Fig. 2E).

### An optimized formulation enhanced oral bioavailability.

Pharmacokinetic studies in Swiss Webster mice were performed with compound 2 dosed by oral and intravenous (i.v.) injection. An oral propylene glycol (PG):Tween 80 formulation was compared with a carboxymethyl cellulose (CMC) formulation at the 10 mg/kg of body weight dosing level. The optimized PG:Tween 80 formulation significantly improved the pharmacokinetic parameters compared with the universal 0.5% (wt/vol) CMC formulation, as evidenced by improved maximum concentration of drug in serum (*C*_max_) (1.04 versus 0.15 μg/ml) and area under the curve to the last measurable concentration (AUC_last_) (6.56 versus 1.51 h·μg/ml) (Table 1). The optimized formulation provided a relative bioavailability of 96%, which represents a 74% increase compared with the CMC formulation (22%) (Table 1). Other formulations, such as 0.5% wt/vol methylcellulose nano-suspension and solid dispersion, were also tested for comparison, but all were inferior to the PG:Tween 80 formulation (see Table S1 in the supplemental material). The plasma concentration of free compound 2 exceeded the MIC (0.004 μg/ml) for 24 h postdose with both the oral (p.o.) and i.v. dosing regimens (see Fig. S1 in the supplemental material). The plasma protein binding of compound 2 in mouse was 96.8%.

**TABLE 1:** Single dose^*a*^ pharmacokinetics of compound 2^*b*^

Parameter	Values by formulation	
	0.5% CMC	PG:Tween 80
*T*_max_ (h)	1.25 ± 1.06	1.00 ± 0.00
*C*_max_ (μg/ml)	0.15 ± 0.04	1.04 ± 0.02
AUC_last_ (h·μg/ml)	1.51 ± 0.56	6.56 ± 0.20
AUC_inf_ (h·μg/ml)	1.55 ± 0.56	9.03 ± 0.26
F (%)	22	96

a 10 mg/kg, p.o.

b Data are mean ± SD unless otherwise indicated. *T*_max_, time to maximum concentration of drug in serum. AUC_inf_, area under the curve extrapolated to infinity.

### Improved *in vivo* efficacy of the optimized formulation in a mouse infection model.

**FIG 3:**
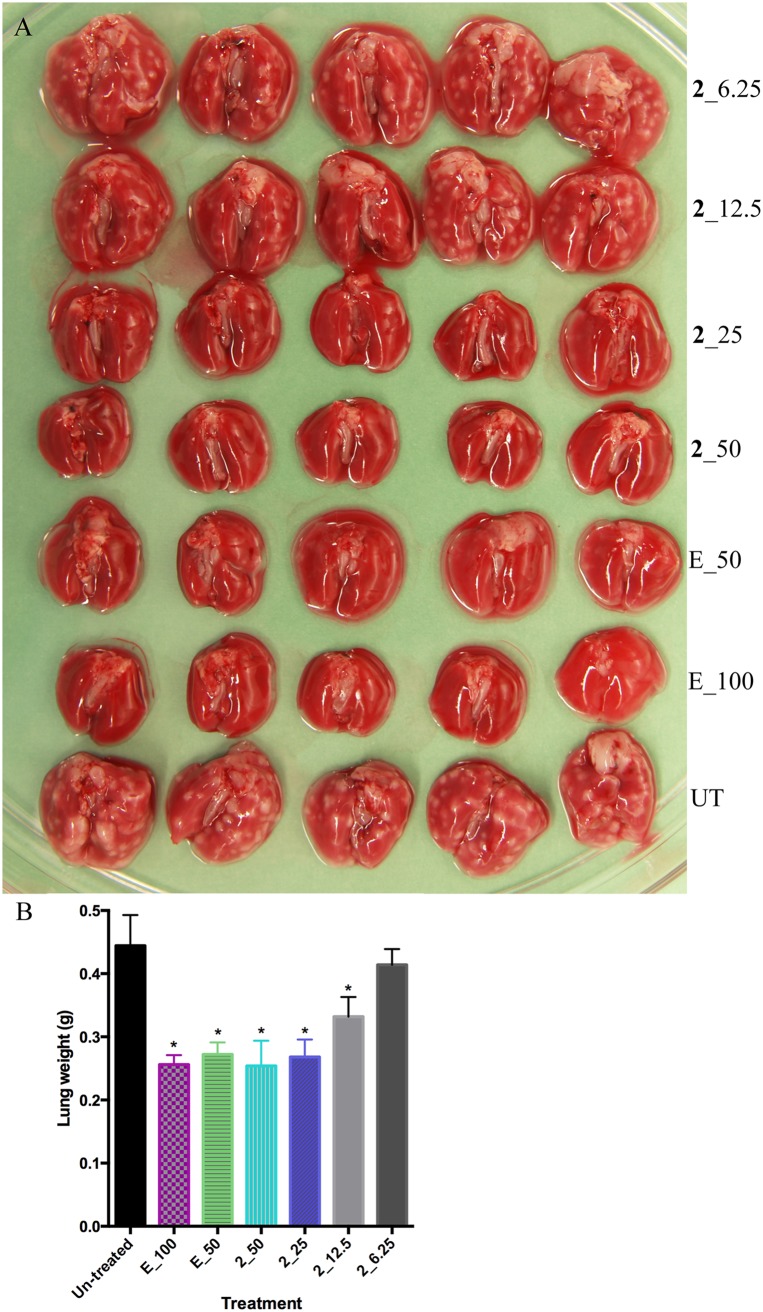
*In vivo* activity of compound 2. Gross pathology (A) and weight (B) of lungs after 4 weeks of treatment. Lung weight was compared with untreated control (*P < 0.0001 by one-way ANOVA and Dunnett’s posttest).

Compound 2 was shown to be active *in vivo* in our previous study (19). We hypothesized that compound 2 prepared in the optimized formulation of PG:Tween 80 (4:1, vol/vol) would have superior activity. Indeed, substantial dose-dependent bactericidal activity was demonstrated by lung CFU counts (Table 2), reduced granuloma formation (Fig. 3A), and lung weight (Fig. 3B). At 50 mg/kg, 4 weeks of treatment with compound 2 achieved a 4.66-log lower mean CFU count than that of untreated controls and 1.2-log lower CFU count than that of the positive control ethambutol at 100 mg/kg. These findings represent a significant improvement compared with the previous study using the 0.5% (wt/vol) CMC formulation, in which 4 weeks of treatment with 100 mg/kg of compound 2 resulted in CFU counts that were 2.0 log higher than the same positive control (19).

**TABLE 2:** Lung CFU counts by week of treatment^*a*^

Treatment	Values by treatment time point		
	Day 1	Week 2	Week 4
UT	2.35 (0.19)	6.76 (0.04)	7.48 (0.15)
E_100		3.56 (0.26)^*b*^	4.02 (0.28)^*b*^
E_50		4.34 (0.25)^*b*^	5.60 (0.24)^*b*^
2_50		3.40 (NA)^*b*^^,^^*c*^	2.82 (0.34)^*b*^
2_25		3.79 (0.25)^*b*^	5.30 (0.20)^*b*^
2_12.5		5.31 (0.48)^*b*^	6.63 (0.21)^*b*^
2_6.25		6.65 (0.20)	7.11 (0.10)

a All values are mean (±SD).

b Significantly different from untreated control (UT) (*P* < 0.0001).

c No standard deviation because of a single value.

### Superior *in vivo* efficacy of compound 2 compared with compound 3.

**FIG 4:**
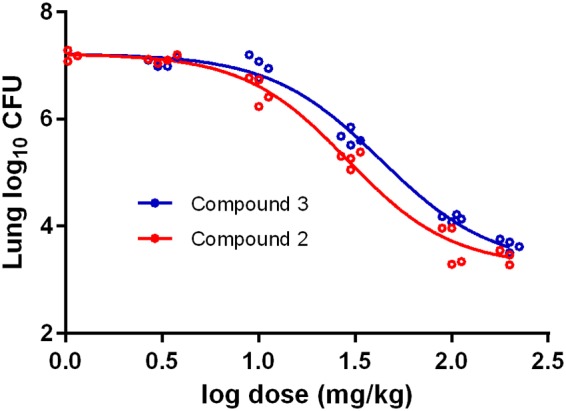
Comparative dose-response curves for compounds 2 and 3 in a high-dose aerosol mouse infection model (*P* < 0.0001 for comparison of log ED_50_ by extra sum-of-squares F test).

To compare the *in vivo* efficacy of compound 2 with that of the published indoleamide analog compound 3 (17), we evaluated their dose-ranging activity in a mouse high-dose aerosol infection model. Treatment commenced 2 weeks after implantation of 3.72 log_10_ CFU of M. tuberculosis H37Rv, when the mean lung CFU count was 6.27 log_10_. Both compounds showed dose-dependent activity (Fig. 4), with bactericidal effects observed at doses of ≥30 mg/kg (*P* < 0.01) (see Table S2 in the supplemental material). At 10 mg/kg, compound 2 significantly reduced the lung CFU burden compared with untreated controls (*P* < 0.05), but compound 3 did not. On comparison of the sigmoidal dose-response curves, the 50% effective dose (ED_50_) was statistically significantly lower for compound 2 (28.9; 95% confidence interval [CI], 25.0 to 33.4) than for compound 3 (43.0; 95% CI, 36.9 to 50.1) (*P* < 0.0001), indicating the superior *in vivo* potency of compound 2 (Fig. 4; Table S2).

## DISCUSSION

MmpL3 is an emerging target for new antituberculosis drugs. Indoleamide inhibitors of MmpL3 previously demonstrated *in vitro* and *in vivo* activity warranting further development of this scaffold (10, 11, 17–19). In this study, we observed the effect of indoleamide treatment on M. tuberculosis cell morphology *in vitro*. Dimples on the poles and septum and a wrinkled cell surface near the poles were induced in a dose-dependent manner, consistent with the findings by Carel et al. in a mycobacterial model system that MmpL3 specifically and dynamically accumulated at the poles and septa during bacterial growth (20). Moreover, the dynamic colocalization of MmpL3 with Wag31, the protein responsible for polar localization of mycobacterial peptidoglycan biosynthesis, suggests that the main components of the mycomembrane may potentially be synthesized at these precise loci (20). Wag31 proteomic interactome pulldown analysis identified MmpL3, among five other proteins, as a binding partner, and implicated MmpL3 in cell wall lipid permeability and multiple lipophilic antibiotic resistance (21). These characteristics justify MmpL3 as a potential therapeutic target for novel inhibitors, such as indoleamides.

We optimized the formulation for oral administration and reevaluated a representative expanded-spectrum indoleamide, compound 2, achieving significant improvements in bioavailability and *in vivo* efficacy. Compound 2 is insoluble in water with a predicted partition coefficient (clogP) of 5.74. In our previous study using the universal 0.5% (wt/vol) CMC formulation, compound 2 showed therapeutic efficacy in a mouse infection model, but dosing at 100 mg/kg did not achieve efficacy comparable to the ethambutol control (19). In the current study, a 50-mg/kg dose in the optimized formulation achieved far superior *in vivo* efficacy compared with the same ethambutol control, demonstrating the *in vivo* bactericidal potential of this class. Because of the suboptimal *in vivo* activity of compound 2 in previous work (19), we used bacteriostatic ethambutol rather than bactericidal INH as a positive control in the current study. Although monotherapy with 50 mg/kg of compound 2 did not lower the lung CFU burdens in the day 1 model, bactericidal activity (log CFU reduction) was achieved in the day 14 acute model for both compound 2 and compound 3. This could be attributable to higher dose levels (100 and 200 mg/kg) and the impact of the host adaptive immune response.

Two independent groups have developed and characterized indoleamides as a scaffold targeting MmpL3 of M. tuberculosis *in vitro* and *in vivo* simultaneously (10, 11, 17, 18). In this study, we compared two expanded-spectrum indoleamide lead compounds from these two programs, compound 3 and compound 2, side-by-side in a mouse infection model. While both compounds exhibited bactericidal activity at doses of 30 mg/kg and above, compound 2 showed statistically significantly greater potency (i.e., lower ED_50_) than compound 3, suggesting that, if similar exposures of the two compounds could be safely attained in humans, then compound 2 should be more active.

Indoleamides are active not only on drug-susceptible but also on drug-resistant M. tuberculosis (17, 18). Most interestingly, indoleamides showed synergy with the first-line TB drug rifampin in a mouse infection model (19). In addition, the spontaneous resistance frequency is rather low (17, 18). The improved efficacy demonstrated with the optimized formulation improvement in this study makes this structural class even more promising for further development as a novel component in new regimens to treat drug-susceptible and drug-resistant TB.

## MATERIALS AND METHODS

### Mycobacterial strain and culture conditions.

M. tuberculosis H37Rv was cultured in 7H9 broth supplemented with 0.2% glycerol, 10% oleic acid-albumin-dextrose-catalase (OADC) (Becton, Dickinson, USA), and 0.05% Tween 80. CFUs were determined by plating diluted mouse lung homogenates onto 7H11 agar containing 50 μg/ml cycloheximide, 25 μg/ml polymixin B, 50 μg/ml carbenicillin, and 20 μg/ml trimethoprim. Incubation was carried out at 37°C.

### Field emission scanning electron microscopy.

To study the effect of indoleamide on mycobacterial cell morphology, M. tuberculosis H37Rv was cultured to optical density at 600 (OD_600_) of 0.06. Compound 1 (Fig. 1) (18) was added to a final concentration of 0.004 (1× MIC) or 0.04 μg/ml (10× MIC). Isoniazid (0.4 μg/ml) was used as the control. Samples were collected after 24 h of incubation. Bacteria were immobilized to poly-l-lysine-charged coverslips for 30 min and processed for field emission scanning electron microscopy (FESEM), as described previously (22). For quantification, 100 cells from at least 10 fields from each of three biological replicates were counted under a fixed magnification.

### Pharmacokinetic study.

To enhance oral bioavailability, we developed a novel formulation for compound 2 in propylene glycol (PG):Tween 80 (4:1, vol/vol). We compared the bioavailability of this new formulation with a previously reported formulation using 0.5% (wt/vol) carboxymethyl cellulose (CMC) (19). For oral (p.o.) administration, compound 2 was prepared in either formulation at 1 mg/ml of homogeneous suspension to achieve a 10-mg/kg dose level. For intravenous (i.v.) injection, a 0.22-mg/ml solution of compound 2 in PG:Tween 80:5% glucose at 20:10:70 (vol/vol/vol) was used. Five-week-old male Swiss Webster mice were randomly assigned to time points (3 mice per arm per time point). Serial blood samples of approximately 30 μl were collected at 0.25, 0.5, 1, 2, 4, 8, and 24 h after a single p.o. dose; and at 0.083, 0.33, 1, 2, 4, 8, and 24 h after a single i.v. dose. Plasma was harvested and stored at −80°C until analysis. Plasma samples were analyzed for compound 2 using liquid chromatography-tandem mass spectrometry (LC-MS/MS), with a limit of quantification of 1.05 ng/ml. Estimation of pharmacokinetic parameters was conducted using noncompartmental analysis with WinNonlin software (Phoenix 6.3).

### *In vivo* efficacy evaluation.

Four- to 6-week-old female BALB/c mice were obtained from Charles River Laboratories. Mice were aerosol infected with M. tuberculosis H37Rv using an inhalation system (Glas-Col Inc., Terre Haute, IN). At day 1 and day 3 postinfection, 3 mice were sacrificed to determine the number of CFU implanted and the pretreatment CFU burden in the lungs, respectively. From day 3 after infection, groups of 5 mice were treated with 6.25, 12.5, 25, or 50 mg/kg of compound 2 by daily gavage (5 days per week). Ethambutol was administered at 100 and 50 mg/kg to positive controls. Infected but untreated mice were used as negative controls. Compound 2 was prepared with the improved PG:Tween 80 (4:1 vol/vol) vehicle. At days 14 and 28 after treatment initiation, 5 mice from each treatment group were sacrificed and the lungs removed. At the latter time point, lungs were photographed for gross pathology comparison. Lungs were homogenized, diluted, and plated onto 7H11 selective agar plates to enumerate CFUs.

To compare the *in vivo* efficacy and dose-response relationships of compound 2 and compound 3 (Fig. 1) (17), female BALB/c mice, 5 weeks of age, were aerosol infected with approximately 5,000 CFUs of M. tuberculosis H37Rv. Treatment started 2 weeks after infection. Both compounds were prepared in a lipid-based microemulsion preconcentrate (MEPC) formulation previously developed for NITD-304 (17). Doses ranged from 3, 10, 30, 100, to 200 mg/kg. Mice were dosed daily gavage (5 days/week) for 4 weeks. Five mice per treatment group were sacrificed after 4 weeks of treatment for lung CFU enumeration.

The Institutional Animal Care and Use Committee of the Johns Hopkins University School of Medicine approved all animal procedures performed in this study.

### Statistical analysis.

Lung CFU counts (x) were log transformed (as x + 1) before analysis. Group mean CFU counts were compared using one-way analysis of variance (ANOVA) with Dunnett’s posttest to adjust for multiple comparisons. Dose-response curves were fit by nonlinear regression using a four-parameter inhibitory sigmoidal maximum effect (*E*_max_) equation, assuming the same *E*_max_ for both compounds. ED_50_ values were compared using the extra sum-of-squares F test. All analyses were performed with GraphPad Prism version 6 (GraphPad, San Diego, CA).

## Supplementary Material

Supplemental file 1
