# Supplementary material for: Advancing the Therapeutic Potential of Indoleamides for Tuberculosis
Source: Antimicrob Agents Chemother. 2019 Jun 24;63(7):e00343-19. doi: 10.1128/AAC.00343-19 (PMC6591635; doi:10.1128/AAC.00343-19)
Supplement: Supplemental file 1 [file AAC.00343-19-s0001.pdf]

Table S1. Plasma PK parameters of JS-2-02 in different formulations in mouse (n=3)

Parameters	ORAL FORMULATIONS			
	0.5% w/v methylcellulose	0.5% w/v methylcellulose nano-suspension	Solid dispersion	PG: Tween 80 Suspension
Dose (mg/kg)	10	10	10	10
F (%)	22	24	7	96

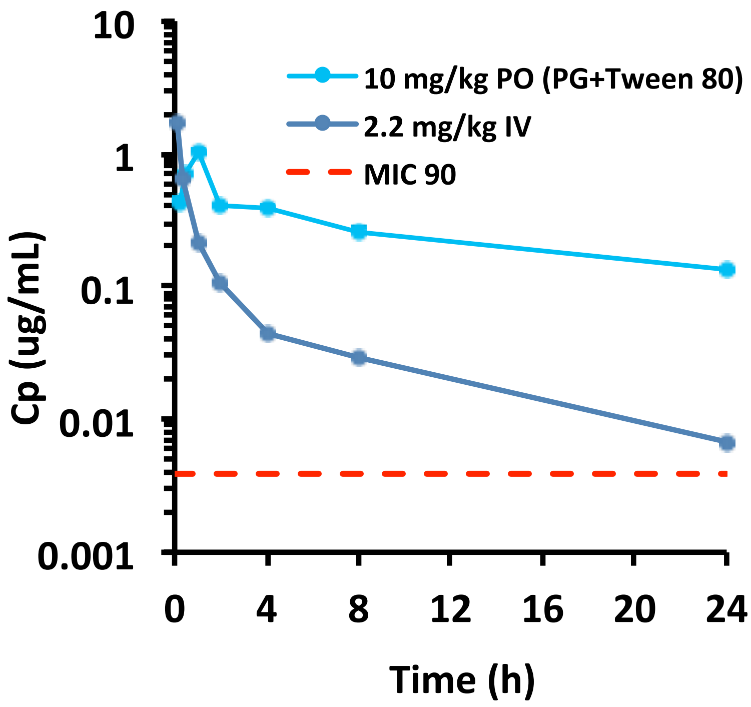

Fig. S1. Plasma concentration of compound **2** after single dose administration

Table S2. Comparative dose ranging in acute mouse infection and monotherapy model

	**D-13**	**D0**	**D28**		
			UT	compound2	compound3
UT	3.72±0.02	6.27±0.10	7.21±0.10		
3				7.11±0.07	7.05±0.09
10				6.54±0.26	6.99±0.19
30				5.25±0.14	5.66±0.14
100				3.64±0.37	4.15±0.06
200				3.43±0.14	3.65±0.11
